# Comparison of volume-controlled ventilation mode and pressure-controlled ventilation with volume-guaranteed mode in the prone position during lumbar spine surgery

**DOI:** 10.1186/s12871-019-0806-7

**Published:** 2019-07-27

**Authors:** Jung Min Lee, Soo Kyung Lee, Kyung Mi Kim, You Jung Kim, Eun Young Park

**Affiliations:** Department of Anesthesiology and Pain Medicine, Hallym University Sacred Heart Hospital, College of Medicine, Hallym University, 22, Gwanpyeong-ro 170beon-gil, Dongan-gu, Gyeonggi-do, 14068 Anyang-si, Republic of Korea

**Keywords:** Mechanical ventilation, General anesthesia, Spine surgery, Cardiac output

## Abstract

**Background:**

During lumbar spine surgery, patients are placed in the prone position for surgical access. The prone position has various effects on cardiac and pulmonary function, including a decreased cardiac index (CI), decreased dynamic lung compliance (Cdyn), and increased peak inspiratory pressure (Ppeak). In this study, we compared the volume-controlled ventilation mode (VCV) and pressure-controlled ventilation with volume guaranteed mode (PCV-VG) based on hemodynamic and pulmonary variables in the prone position during lumbar spine surgery.

**Methods:**

Thirty-six patients scheduled for lumbar spine surgery in the prone position were enrolled in this prospective, randomized clinical trial. The patients were randomly assigned to receive VCV or PCV-VG. Hemodynamic variables, respiratory variables, and arterial blood gases were measured in the supine position 15 min after the induction of anesthesia, 15 min after placement in the prone position, 30 min after placement in the prone position, and 15 min after placement in the supine position at the end of anesthesia.

**Results:**

The hemodynamic variables and arterial blood gas results did not differ significantly between the two groups. Lower Ppeak values were observed in the PCV-VG group than in the VCV group (*p* = 0.045). The Cdyn values in the VCV group were lower than those in the PCV-VG group (*p* = 0.040).

**Conclusion:**

PCV-VG led to lower Ppeak and improved Cdyn values compared with VCV, showing that it may be a favorable alternative mode of mechanical ventilation for patients in the prone position during lumbar spine surgery.

**Trial registration:**

The study was retrospectively registered at ClinicalTrials.gov (NCT 03571854). The initial registration date was 6/18/2018.

## Background

The prone position is commonly required to enable surgical access during lumbar spine surgery. When a patient moves into the prone position, inferior vena cava obstruction causes reduced venous return and increased thoracic pressure results in reduced left ventricular compliance, leading to a decreased cardiac index (CI) [1]. Pulmonary physiology is also influenced in the prone position. Although the V/Q mismatch is reduced and arterial oxygenation is improved in the prone position [2], lung compliance is reduced and the peak inspiratory pressure (Ppeak) increases to achieve the set tidal volume [3].

The volume-controlled ventilation mode (VCV) is commonly used during general anesthesia: the minute ventilation is fixed and airway pressure is influenced by pulmonary resistance or compliance [4]. The pressure-controlled ventilation with volume guaranteed mode (PCV-VG) has recently been introduced in the field of anesthesiology. In this mode, the ventilator compares the tidal volume of the previous breath and automatically regulates the pressure limits to achieve a set tidal volume with the lowest airway pressure [5, 6].

In this randomized study, we tested the null hypothesis that VCV or PCV-VG does not affect hemodynamic or pulmonary variables during lumbar spine surgery in the prone position. The primary outcome of this study was Ppeak. The secondary outcomes were lung compliance and hemodynamic variables including cardiac output (CO), CI, stroke volume (SV), and stroke volume variation (SVV).

## Methods

This study was approved by the Institutional Review Board of Hallym University Sacred Heart Hospital. After written informed consent was obtained from all participants, 36 patients who were scheduled for lumbar spine surgery in the prone position were enrolled. Patients with morbid obesity (body mass index > 30 kg/m^2^), hypotension (systolic blood pressure < 100 mmHg), bradycardia (heart rate < 60 bpm), uncompensated cardiologic disease (heart failure, history of myocardial infarction, or heart block), hypoxia (PaO_2_ < 60 mmHg or SpO_2_ < 90%), uncontrolled asthma, or chronic obstructive pulmonary disease (forced expiratory volume in 1 s < 60%) were excluded. Patients who were younger than 20 years or older than 70 years were also excluded. This study was registered at ClinicalTrials.gov (NCT 03571854). The study was conducted in accordance with the Consolidated Standards of Reporting Trials (CONSORT) 2010 statement [7].

All patients fasted for 8 h before surgery and were premedicated with intramuscular glycopyrrolate (0.2 mg). In the operating room, patients were monitored by non-invasive blood pressure, electrocardiography, and pulse oximetry (SpO_2_) before the induction of anesthesia. Anesthesia was induced with remifentanil (0.1–0.2 μg/kg/min), propofol (1.5–2 mg/kg), and rocuronium (0.6 mg/kg), and maintained at a fractional inspired oxygen concentration of 0.5 with sevoflurane (2.0–2.5 vol%), remifentanil (0.05–0.3 μg/kg/min), and vecuronium (0.03–0.05 mg/kg/h). The patients were ventilated with a Datex-Ohmeda Ventilator (S/S AVANCE) and randomly assigned to receive either VCV (*n* = 18) or PCV-VG (n = 18) using a computer-generated randomization method. The tidal volume in both groups was set to deliver 8 mL/kg of ideal body weight. The respiratory rate (RR) was adjusted to maintain an end tidal CO_2_ (ETCO_2_) level of 33–38 mmHg, and the inspiratory to expiratory time (I:E) ratio was 0.5. After the induction of anesthesia, a 20 G catheter was inserted into the radial artery to monitor continuous arterial pressure and connected to the FloTrac®/Vigileo system (Edwards Lifesciences Corp., Irvine, CA) for the continuous monitoring of variables, including CO, CI, SV, stroke volume index (SVI), and SVV.

Hemodynamic variables, respiratory variables, and arterial blood gases were measured in the supine position 15 min after the induction of anesthesia, 15 min after placement in the prone position, 30 min after placement in the prone position, and 15 min after placement in the supine position at the end of anesthesia. The measured hemodynamic variables included mean arterial pressure (MAP), heart rate (HR), CO, CI, SV, SVI, and SVV. Respiratory variables included RR, Ppeak, mean inspiratory pressure (Pmean), SpO_2_, ETCO_2_, and dynamic lung compliance (Cdyn).

As there have been no previous studies comparing the effects of PCV-VG and VCV during surgery in the prone position, the sample size for this study was determined in a pilot study. The results indicated a mean difference in Ppeak between the two modes of 10% (α = 0.05, power = 80%, effect size = 1.007). Assuming a 20% dropout rate, 18 patients were included in each group in this study.

All data are presented as the median (interquartile range) or number of patients. Hemodynamic and respiratory data were analyzed with a repeated-measures analysis of variance, and differences between groups at the same time point were analyzed with the two-sample *t*-test or Mann-Whitney U test; *p*-values < 0.05 were considered statistically significant. Statistical analyses were performed using the Statistical Package for the Social Sciences version 24.0 (IBM Corp., Armonk, NY).

## Results

**Fig. 1:**
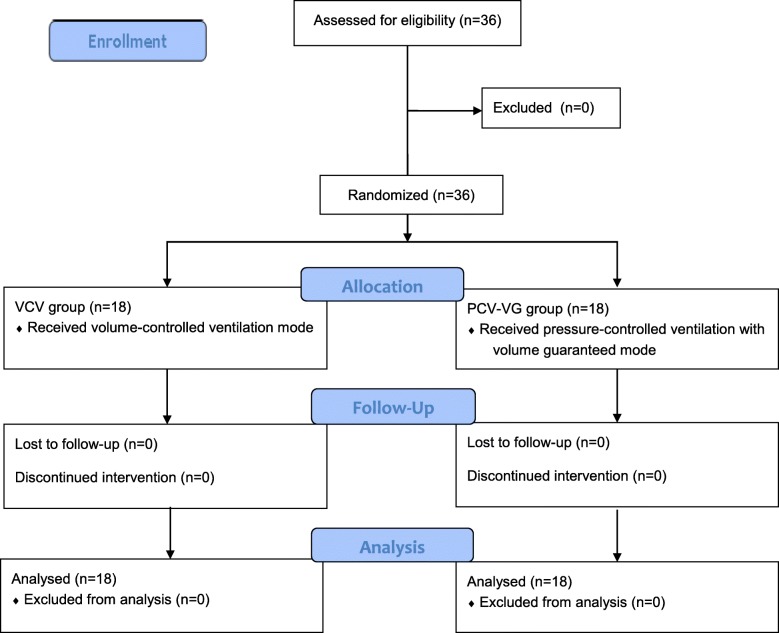
The CONSORT flow diagram

**Fig. 2:**
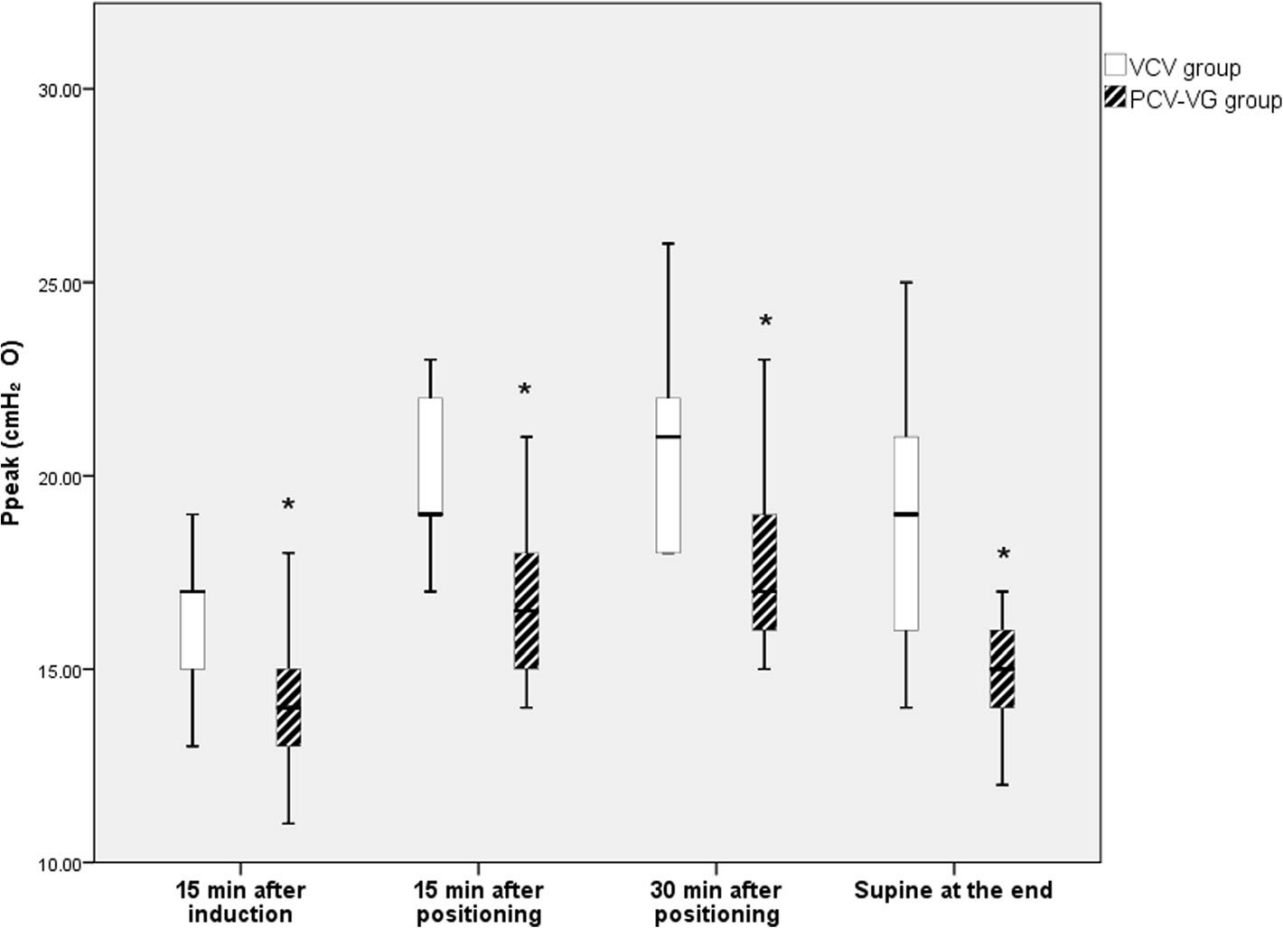
Peak airway pressure at each measurement timepoint in each group Data are displayed as ranges, medians, and interquartile ranges. VCV, volume controlled ventilation; PCV-VG, pressure-controlled ventilation with volume guaranteed mode. *p < 0.05 compared with the VCV group at the same time point

**Fig. 3:**
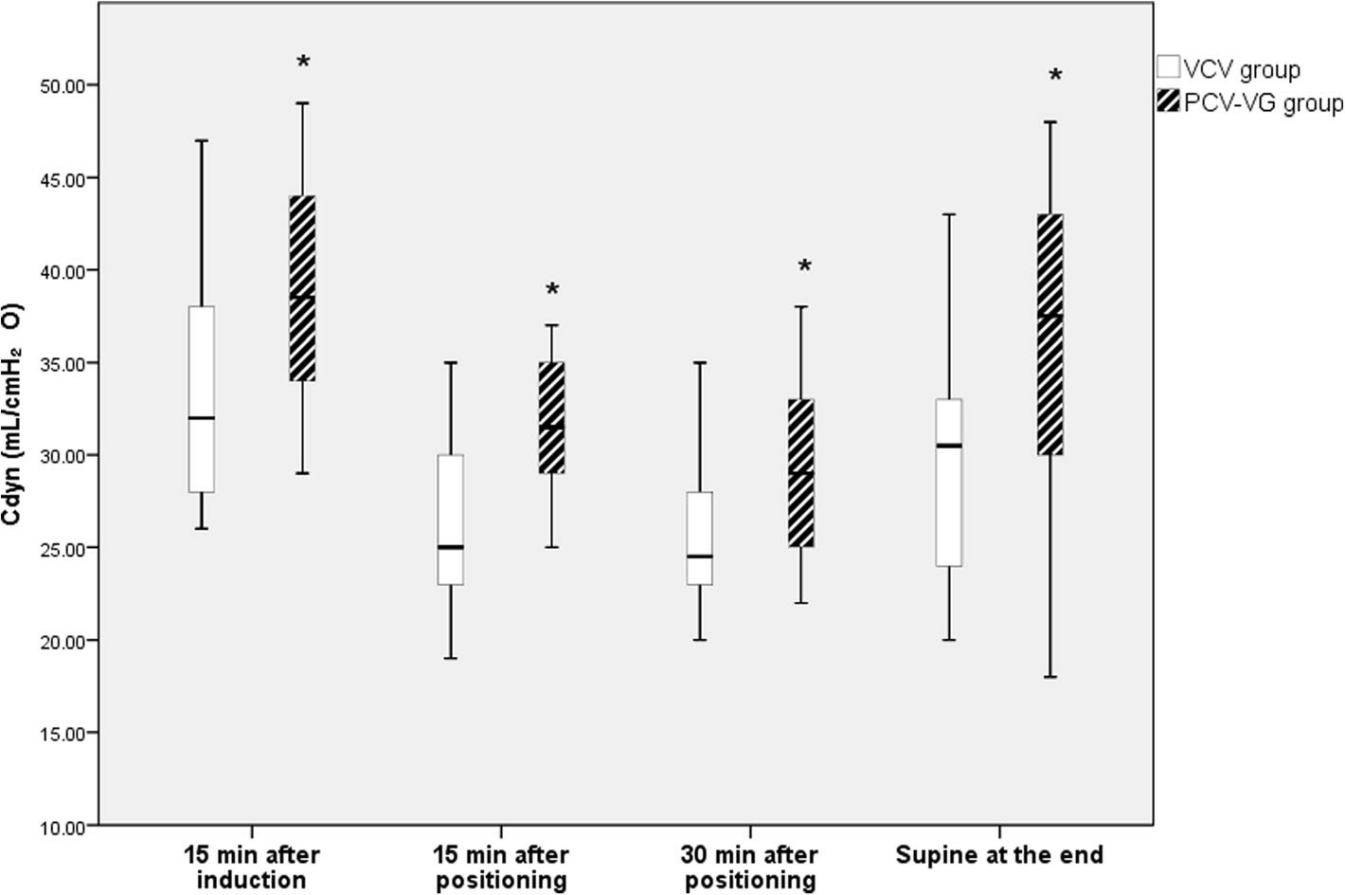
Dynamic lung compliance at each measurement timepoint in each group. Data are displayed as ranges, medians, and interquartile ranges. VCV, volume-controlled ventilation, PCV-VG, pressure-controlled ventilation with volume guaranteed. *p < 0.05 compared with the VCV group at the same time point

A total of 36 patients were enrolled in this study and a CONSORT flow diagram is shown in Fig. 1. There were no significant differences between the groups in patient characteristics (Table 1). Among the respiratory variables, Ppeak increased after placement in the prone position during surgery and in the supine position at the end of anesthesia in both groups, and there were lower Ppeak values in the PCV-VG group than in the VCV group throughout the study period (*p* = 0.045, Fig. 2). The Cdyn values at the later three time points were lower than the initial values in both groups, and Cdyn was lower in the VCV group than in the PCV-VG group throughout the study period (*p* = 0.040, Fig. 3). Pmean increased after placement in the prone position in both groups, and there was no significant intergroup difference in Pmean over time during surgery (Table 2).

**Table 1:** Patient characteristics

	VCV (*n* = 18)	PCV-VG (*n* = 18)
Age (years)	50.0 (41–60)	56 (52–61)
Male/female	11/7	11/7
BMI (kg/m^2^)	24.1 (22.4–26.8)	24.5 (22.6–27.4)
ASA PS classification (I/II/III)	5/9/4	2/13/3
Duration of anesthesia (min)	240 (176–280)	295 (204–341)
Duration of surgery (min)	163 (108–196)	223 (115–244)

*BMI* body mass index, *ASA PS* classification, American Society of Anesthesiologists physical status classification, *VCV* volume-controlled ventilation, *PCV-VG* pressure-controlled ventilation with volume guaranteed

Data are listed as the median (interquartile range) or number of patients

**Table 2:** Respiratory variables

	Group	15 min after induction	15 min after positioning	30 min after positioning	Supine at the end
Ppeak (cmH_2_O)	VCV	17 (15–17)	19 (19–22)†	21 (18–22)†	19 (16–22)†
	PCV-VG	14 (13–15)*	17 (15–18)*†	17 (16–19)*†	15 (14–16)*
Pmean (cmH_2_O)	VCV	7 (6–7)	7 (7–8)†	8 (7–8)†	7 (6–8)
	PCV-VG	7 (6–7)	7 (7–8)†	8 (7–9)†	7 (7–8)
RR (breaths/min)	VCV	12 (12–14)	11 (10–12)	12 (10–12)	12 (10–13)
	PCV-VG	12 (11–13)	12 (10–12)	12 (10–12)	11 (10–13)
Cdyn (mL/cmH_2_O)	VCV	32 (28–39)	25 (23–30)†	25 (23–28)†	31 (24–33)†
	PCV-VG	39 (34–44)*	32 (29–35)*†	29 (25–33)*†	38 (30–43)*†
ETCO_2_ (mmHg)	VCV	33 (31–34)	32 (30–33)	32 (30–35)	34 (31–35)
	PCV-VG	31 (30–33)	32 (31–34)	32 (30–34)	33 (31–35)
SaO_2_ (%)	VCV	99 (98–100)	99 (98–99)	99 (98–100)	99 (99–100)
	PCV-VG	99 (98–100)	99 (98–100)	99 (98–100)	99 (99–100)

*Ppeak* peak inspiratory pressure, *Pmean* mean inspiratory pressure, *RR* respiratory rate, *Cdyn* dynamic compliance, *ETCO*_*2*_ end tidal CO_2_, *VCV* volume-controlled ventilation, *PCV-VG* pressure-controlled ventilation with volume guaranteed

Data are listed as the median (interquartile range)

*p < 0.05 compared with the VCV group at the same time point

†*p* < 0.05 compared with 15 min after induction in each group

HR decreased after placement in the prone position in both groups, but no significant intergroup difference was present. CO and CI did not differ significantly between groups, while the CO and CI values decreased 15 min after placement in the prone position (Table 3). The blood gas results did not differ between groups (Table 4).

**Table 3:** Hemodynamic variables

	Group	15 min after induction	15 min after positioning	30 min after positioning	Supine at the end
MAP (mmHg)	VCV	89 (80–100)	86 (77–98)	82 (75–90)	106 (88–109)
	PCV-VG	92 (71–102)	83 (72–93)	82 (73–94)	91 (80–98)
HR (beats/min)	VCV	78 (65–82)	68 (56–71)†	66 (61–69)†	71 (65–89)
	PCV-VG	70 (63–73)	63 (58–69)†	61 (56–67)†	73 (63–93)†
CO (L/min)	VCV	4.7 (3.8–5.7)	4.0 (3.2–4.7)†	4.1 (3.2–4.8)	5.4 (3.7–7.3)
	PCV-VG	4.2 (3.7–4.4)	3.6 (3.3–4.0)†	3.7 (3.3–4.2)	4.6 (4.0–5.7)†
CI (L/min/m^2^)	VCV	2.8 (2.2–3.1)	2.3 (1.9–2.7)†	2.4 (1.7–2.9)	3.2 (2.4–3.3)
	PCV-VG	2.4 (2.2–2.6)	2.3 (1.8–2.4)†	2.2 (1.9–2.6)	2.8 (2.4–3.3)†
SV (mL/beat)	VCV	63 (54–75)	56 (46–74)	57 (47–71)	67 (56–84)
	PCV-VG	57 (55–65)	59 (51–66)	59 (53–65)	66 (54–78)
SVI (mL/m^2^/beat)	VCV	37 (32–41)	33 (29–39)	34 (26–40)	38 (32–51)
	PCV-VG	34 (32–39)	34 (31–40)	36 (32–40)	39 (32–44)
SVV (%)	VCV	11 (9–14)	12 (10–16)	11 (9–17)	10 (6–13)
	PCV-VG	12 (8–14)	13 (10–15)	13 (10–15)	9 (8–15)

*MAP* mean arterial pressure, *HR* heart rate, *CO* cardiac output, *CI* cardiac index, *SV* stroke volume, *SVI* stroke volume index, *SVV* stroke volume variation, *VCV* volume controlled ventilation, *PCV-VG*, pressure controlled ventilation with volume guaranteed

Data are listed as the median (interquartile range)

†*p* < 0.05 compared with 15 min after induction in each group

**Table 4:** Arterial blood gas analyses

	Group	15 min after induction	15 min after positioning	30 min after positioning	Supine at the end
pH	VCV	7.44 (7.42–7.45)	7.44 (7.41–7.47)	7.44 (7.42–7.46)	7.42 (7.38–7.45)
	PCV-VG	7.43 (7.41–7.48)	7.43 (7.42–7.45)	7.42 (7.40–7.47)	7.41 (7.39–7.45)
PaO_2_ (mmHg)	VCV	242 (209–285)	252 (237–271)	258 (234–272)	216 (192–255)
	PCV-VG	209 (177–248)	234 (215–260)	233 (218–259)	230 (215–262)
PaCO_2_ (mmHg)	VCV	32 (30–34)	31 (30–33)	31 (30–33)	34 (31–36)
	PCV-VG	34 (31–35)	33 (31–35)	34 (31–35)	35 (32–38)
SaO_2_ (%)	VCV	99.9 (99.8–99.9)	99.9 (99.8–99.9)	99.9 (99.8–99.9)	99.8 (99.7–99.9)
	PCV-VG	99.8 (99.6–99.9)	99.9 (99.8–99.9)	99.8 (99.8–99.9)	99.8 (99.8–99.9)

*PaO*_*2*_ partial arterial oxygen tension, *PaCO*_*2*_ partial arterial carbon dioxide tension, *VCV* volume-controlled ventilation, *PCV-VG* pressure-controlled ventilation with volume guaranteed

Data are listed as the median (interquartile range)

## Discussion

In the present study, we compared the effects of the VCV and PCV-VG on hemodynamic and pulmonary variables in the prone position. In VCV, the ventilator delivers a target volume with a constant flow and the airway pressure increases in a linear manner. This approach ensures minute ventilation regardless of airway compliance but cannot control airway pressure [8]. Compared with VCV, the pressure-controlled ventilation mode (PCV) provides the tidal volume at a preset pressure with decelerating flow, and the tidal volume can be varied depending on lung compliance [9]. PCV-VG delivers a target tidal volume with a decelerating flow and calculates lung compliance to adjust the inspiratory pressure based on the previous breath [6, 8]. It reaches the target volume with the lowest inspiratory pressure and has the benefits of both VCV and PCV [9].

Several studies have examined the effects of the PCV-VG compared with conventional modes (VCV or PCV). In thoracic surgery with one-lung ventilation, PCV-VG led to lower Ppeak, peak plateau pressure, and Pmean values compared with VCV [10, 11]. Similar results were obtained in a laparoscopic surgery study that showed lower Ppeak values with PCV-VG than with VCV [6, 9, 12].

Spine surgery is commonly performed with patients in the prone position. In respiratory physiology, Ppeak increases and Cdyn decreases as increased intra-thoracic pressure and abdominal pressure compromise diaphragm movement when a patient is turned to the prone position [3, 4, 13, 14]. To our knowledge, no previous study has compared VCV and PCV-VG in the prone position. However, several studies have compared PCV and VCV for patients undergoing posterior lumbar surgery and found that PCV is associated with lower Ppeak values compared with VCV [13, 14].

Pmean correlates with alveolar ventilation and improved oxygenation [15]. Although the values for patients in the prone position were higher than for patients in the supine position in both groups, placement in the prone position did not significantly improve oxygenation. One previous study found similar results; the authors attributed them to a lack of positive end-expiratory pressure [14].

It is known that the CI decreases when a patient moves in the prone position. This is caused by reduced venous return and increased intra-thoracic pressure, which results in decreased arterial filling and reduced ventricular compliance [1]. Dharmavaram et al. [16] compared the effects of prone positioners on hemodynamic values using transesophageal echocardiography (TEE). They found that decreases in CI in the prone position resulted from a decreased SV. This effect seemed to be caused by increased afterload rather than decreased preload because the left ventricle end-diastolic area was not significantly altered. Blood flow through the mitral valve was reduced on TEE, suggesting that reduced chest wall compliance diminished diastolic function and increased afterload. The Jackson table had less effect on cardiac function than the Wilson frame.

In the present study, the CO and CI values measured 15 min after placement in the prone position were lower compared with the same values measured in the supine position. The SV values did not significantly change as patients were positioned on the Jackson table, so it is thought that the decreased CO and CI in the prone position may have been caused by a decreased HR, rather than decreased preload or increased afterload.

It can be expected that differences in Ppeak between VCV and PCV-VG will affect pleural pressure, inducing changes in the cardiovascular response [17]. However, we noted no significant hemodynamic change between VCV and PCV-VG. An animal study comparing the effects of VCV and PCV on anesthetized dogs showed that hemodynamic functions such as CO, CI, and SVI did not differ significantly between VCV and PCV, although the Ppeak was higher in VCV than in PCV [18]. Balick-Weber et al. [19] used TEE to compare the effects of VCV and PCV on cardiac function and found that there was no echocardiographic change in right ventricular SV, left ventricular preload, or left ventricle end-systolic wall stress. They concluded that Ppeak was not associated with cardiac function and that the main determinant of right ventricle afterload was transpulmonary pressure rather than Ppeak.

The present study has several limitations. First, although the FloTrac®/Vigileo system is widely used in anesthesia, it is an uncalibrated pulse contour analysis and may be less accurate under conditions such as sepsis or reduced systemic vascular resistance [20]. Grensemann et al. [21] reported that the FloTrac®/Vigileo system had a clinically unacceptable high degree of error in the prone position compared with transpulmonary thermodilution techniques and calibrated pulse contour CO analysis. We believed that the transpulmonary thermodilution technique using a pulmonary catheter would be more invasive than pulse contour analysis. As a calibrated pulse contour analysis device was not available in our center, we used an uncalibrated pulse contour analysis device as a less invasive means of measuring hemodynamic variables. Since patients in the previous study were mechanically ventilated with acute respiratory distress syndrome or acute lung injury and expected to be in a septic condition or undergoing vasopressor treatment, the effect of placement in the prone position on uncalibrated CO analysis in healthy volunteers warrants further evaluation. Second, patients with compromised cardiac or pulmonary diseases, as well as those with morbid obesity, were excluded from the study. Respiratory mechanics or hemodynamics may be affected by various lung conditions and we wanted to standardize lung function among the patients. Although there were statistically significant differences between VCV and PCV-VG for Ppeak and Cdyn, these differences were quite small and may have been more prominent for patients with pulmonary disease or morbid obesity. Additional studies are therefore needed to compare the hemodynamic and respiratory effects of VCV and PCV-VG in patients with uncompensated cardiac and pulmonary function or morbid obesity. Third, we measured Cdyn rather than static lung compliance. Static lung compliance does not depend on inspiratory flow and is influenced by the elastic properties of the lung [22]. However, the inspiratory hold maneuver was not available in the ventilator we used, and there may have been detrimental effects on oxygenation while disconnecting the circuit to measure the plateau pressure.

## Conclusion

In conclusion, PCV-VG provided a lower Ppeak and improved Cdyn compared with VCV, and was not associated with significant differences in CO, CI, SV, SVV, and oxygenation. These results show that PCV-VG may be an effective alternative mode of mechanical ventilation for patients in the prone position during lumbar spine surgery. Additional studies are required to evaluate the effects of Ppeak on postoperative patient outcome.

## Data Availability

The data and materials are available from the corresponding author on reasonable request.

## Abbreviations

Cdyn: Dynamic lung compliance
CI: Cardiac index
CO: Cardiac output
ETCO_2_: End tidal carbon dioxide
PaO_2_: Arterial oxygen partial pressure
PCV: Pressure-controlled ventilation
PCV-VG: Pressure-controlled ventilation with volume guaranteed mode
Pmean: Mean inspiratory pressure
Ppeak: Peak inspiratory pressure
RR: Respiratory rate
SV: Stroke volume
SVI: Stroke volume index
SVV: Stroke volume variation
VCV: Volume-controlled ventilation mode
